# The King is Dead, Long Live the King: Entering A New Era of Stem Cell Research and Clinical Development

**DOI:** 10.1186/1479-5876-9-218

**Published:** 2011-12-20

**Authors:** Thomas Ichim, Neil H Riordan, David F Stroncek

**Affiliations:** 1 MediStem Inc., San Diego, CA, USA; 2 Aidan Foundation, Chandler, AZ, USA; 3 Department of Transfusion Medicine, Clinical Center, National Institutes of Health, Bethesda, MD, USA

## Abstract

In mid November the biopharma industry was shocked by the announcement from Geron that they were ending work on embryonic stem cell research and therapy. For more than 10 years the public image of all stem cell research has been equated with embryonic stem cells. Unfortunately, a fundamentally important medical and financial fact was being ignored: embryonic stem cell therapy is extremely immature. In parallel to efforts in embryonic stem cell research and development, scientists and physicians in the field of adult stem cells realized that the natural role of adult stem cells in the body is to promote healing and to act like endogenous "repair cells" and, as a result, numerous companies have entered the field of adult stem cell therapy with the goal of expanding numbers of adult stem cells for administration to patients with various conditions. In contrast to embryonic stem cells, which are extremely expensive and potentially dangerous, adult cell cells are inexpensive and have an excellent safety record when used in humans. Many studies are now showing that adult stem cells are practical, patient-applicable, therapeutics that are very close to being available for incorporation into the practice of medicine. These events signal the entrance of the field of stem cells into a new era: an era where hype and misinformation no longer triumph over economic and medical realities.

## Editorial

As with any major technological advancement, there is an initial period of excitement and euphoria, followed by a crash, followed by a solid steady advancement leading to life-changing advancement. It seems that the field of stem cell research and clinical development is no different.

In mid November the biopharma industry was shocked by the announcement of Geron, the leader of embryonic stem cell research and therapy, quitting work in this area. This news was greeted by negative comments against the whole industry such as "throwing stem cell research into question" (ABC News) and "why would I want to invest in a space where one of the most promising companies just called it quits?" (San Francisco Business Times). It is our position that this announcement is actually the beginning of a new era in stem cell research and clinical therapy, an era where hype and misinformation no longer triumph over economic and medical realities.

Starting in the early 2000s a publicity campaign by embryonic stem cell advocates managed to equate the public image of all stem cell research as involving embryonic stem cells. A debate quickly ensued that polarized the pro-choice and pro-life communities, with statements made equating to "if you don't support embryonic stem cells, you are not supporting cures for terrible disease such as diabetes, Parkinson's, and Alzheimer's" or conversely, accusations that "stem cell researchers are baby killers". While public attention was captivated by this debate, and various State Governments created billion dollar funds to support embryonic stem cell research, a fundamentally important medical and financial fact was being ignored: embryonic stem cells are extremely immature. To generate adult organ, tissues or cells, the embryonic stem cell must "fast forward" the process of years of maturation in a matter of weeks in order to create financially-valuable products. The second fundamental point is the propensity of embryonic stem cells to form tumors called teratomas. In fact, the scientific definition of an embryonic stem cell is a "cell that forms teratomas when placed in animals". Thus while everyone was arguing about the ethics of embryonic stem cell research, the medical and economic realities were ignored: specifically, it costs too much to create clinical products from these cells. This did not stop the "bubble" from growing. The promise of curing incurable diseases with embryonic stem cells seems to have fixated public attention, creating high valuations for companies in this space.

Since 2006 investigators in academia and industry have also been developing induced pluripotent stem cells as an alternative to embryonic stem cells as starting material for stem cell therapy. Induced pluripotent stem cells can be produced from most adult cells and as a result using induced pluripotent stem cells avoids the emotional polarization associated with embryonic stem cells. Induced pluripotent stem cells can also be produced from a patient's own cells, thus avoiding the potential problem of developing therapies that are Human Leukocyte Antigen (HLA) incompatible. Induced pluripotent stem cells, however, share many of the inherent problems associated with embryonic stem cells, such as teratoma formation, difficulty in inducing cell maturation and high costs associated with their clinical application. Working with induced pluripotent stem cells may, in fact, be more difficult and more expensive than working with embryonic stem cells since adult cells must be reprogrammed to produce induced pluripotent stem cells and this likely must be done in a way that avoids leaving a permanent molecular "foot print" in the induced pluripotent stem cells. While induced pluripotent stem cells have tremendous potential for clinical stem cell therapy, more work is needed and the golden era of induced pluripotent stem cells has not yet arrived.

In parallel to efforts in embryonic and induced pluripotent stem cell research and development, scientists and physicians in the field of adult stem cells realized that the natural role of adult stem cells in the body is to promote healing, or in other words, to act like endogenous "repair cells". After a heart attack or stroke the body activates its own adult stem cells in order to try to heal the damaged tissue. In many cases the adult body does not have enough stem cells to heal major injuries such as heart attacks. So numerous companies have entered the field of adult stem cell therapy with the goal of expanding numbers of adult stem cells for administration to patients with various conditions. In contrast to embryonic stem cells, which are extremely expensive and potentially dangerous, adult cell cells are inexpensive and have an excellent safety record when used in humans.

Given that the difference between adult, embryonic and induced pluripotent stem cells is not known to the general public, this exit of Geron from the field of embryonic stem cells has been erroneously interpreted by many as a "major set back for stem cell research". In contrast, nothing could be further from the truth. Ironically, the same day that Geron announced its exit from this field, the adult stem cell company Mesoblast announced results of a double-blind clinical study showing significant benefit in heart failure patients receiving adult stem cells. This was one of many studies showing that adult stem cells are a practical, patient-applicable, therapeutics that are very close to being available for incorporation into the practice of medicine. From a financial perspective, major pharmaceutical companies have already placed their bets on adult stem cells, including the $1.7 Billion Mesoblast-Cephalon deal last year, the 1.3 billion dollar Osiris-Genzyme deal, and Celgene-Anthrogenesis deal several years ago.

Thus, the field of stem cells is entering a new era: an era in which companies will be judged on clinical and financial practicality, not hype and manipulation of public emotion.

